# Genome-Based Insights into the Production of Carotenoids by Antarctic Bacteria, *Planococcus* sp. ANT_H30 and *Rhodococcus* sp. ANT_H53B

**DOI:** 10.3390/molecules25194357

**Published:** 2020-09-23

**Authors:** Michal Styczynski, Agata Rogowska, Katarzyna Gieczewska, Maciej Garstka, Anna Szakiel, Lukasz Dziewit

**Affiliations:** 1 Department of Environmental Microbiology and Biotechnology, Institute of Microbiology, Faculty of Biology, University of Warsaw, Miecznikowa 1, 02-096 Warsaw, Poland; mstyczynski@biol.uw.edu.pl; 2 Department of Plant Biochemistry, Institute of Biochemistry, Faculty of Biology, University of Warsaw, Miecznikowa 1, 02-096 Warsaw, Poland; a.rogowska@biol.uw.edu.pl (A.R.); szakal@biol.uw.edu.pl (A.S.); 3 Department of Plant Anatomy and Cytology, Institute of Experimental Plant Biology and Biotechnology, Faculty of Biology, University of Warsaw, Miecznikowa 1, 02-096 Warsaw, Poland; kat.gieczewska@biol.uw.edu.pl; 4 Department of Metabolic Regulation, Institute of Biochemistry, Faculty of Biology, University of Warsaw, Miecznikowa 1, 02-096 Warsaw, Poland; garstka@biol.uw.edu.pl

**Keywords:** antarctica, carotenoid, *Planococcus*, *Rhodococcus*, secondary metabolite

## Abstract

Antarctic regions are characterized by low temperatures and strong UV radiation. This harsh environment is inhabited by psychrophilic and psychrotolerant organisms, which have developed several adaptive features. In this study, we analyzed two Antarctic bacterial strains, *Planococcus* sp. ANT_H30 and *Rhodococcus* sp. ANT_H53B. The physiological analysis of these strains revealed their potential to produce various biotechnologically valuable secondary metabolites, including surfactants, siderophores, and orange pigments. The genomic characterization of ANT_H30 and ANT_H53B allowed the identification of genes responsible for the production of carotenoids and the in silico reconstruction of the pigment biosynthesis pathways. The complex manual annotation of the bacterial genomes revealed the metabolic potential to degrade a wide variety of compounds, including xenobiotics and waste materials. Carotenoids produced by these bacteria were analyzed chromatographically, and we proved their activity as scavengers of free radicals. The quantity of crude carotenoid extracts produced at two temperatures using various media was also determined. This was a step toward the optimization of carotenoid production by Antarctic bacteria on a larger scale.

## 1. Introduction

Microbial secondary metabolites are relatively low-molecular-mass products of the secondary metabolism that are usually produced during the late growth phase (i.e., idiophase). Secondary metabolites, like pigments, biosurfactants, antibiotics, and siderophores, are not essential for the growth of their producers; however, they may significantly increase the fitness and viability of organisms under specific environmental conditions [1].

One of the main threats to bacteria in the environment is the presence of solar radiation, which is highly variable over a range of scales and wavelengths. Ultraviolet-B (UV-B) radiation (315–280 nm) leads to direct DNA damage by inducing the production of photoproducts, such as cyclobutane pyrimidine dimers and pyrimidine–pyrimidone photoadducts. On the other hand, as a result of UV-A radiation (400–315 nm), reactive oxygen species (ROS) form in the cell, which further leads to the damage of proteins, lipids, and nucleic acids [2]. The carotenoid pigments produced by bacteria, due to their specific structure and antioxidant properties, are the main agents preventing the harmful effects of UV radiation [3]. In permanently cold environments, such as Antarctica, where the temperature during the year is usually below zero and does not exceed 15 °C, carotenoids play a role in the modulation of the membrane fluidity and protect bacterial cells against disruption from freezing [4,5].

There are many carotenoids of bacterial origin, including β-carotene, astaxanthin, and lycopene, that have found applications in the food industry, cosmetology, aquaculture, medicine, and in other industries [6]. In 2010, the global carotenoid market was valued at around $1 billion with clear upward trends. The dominant carotenoid was β-carotene, with a 25% market share. However, the carotenoid with the highest market price was astaxanthin—at $2000/kg of synthetic and $7000/kg of natural pigment [7]. Importantly, synthetic carotenoids have some advantages over natural carotenoids. First, they show greater stability by limited undesirable oxidation or isomerization. In addition, they are prepared in more easily absorbable forms of colloids or water-soluble emulsions. However, carotenoids of synthetic origin may be contaminated with toxic substances that are used for their production that have a negative impact on human health. Therefore, the efficient production of natural carotenoids is particularly important and desired [5]. The growing market demands for carotenoids of natural origin forces biotechnological companies to look for efficient and alternative solutions to their acquisition.

The greatest challenge in the production of natural carotenoids is the high cost, including the energy outlays for maintaining the optimal growth temperatures of the commonly used mesophilic organisms. Therefore, psychrotolerant producers of bioactive compounds appear to be a promising solution, bringing direct economic benefits [8]. Zero waste and circular economy perspectives directed applied microbiology into the screening of microorganisms capable of producing desired metabolites from waste materials. This is another way to reduce the cost of carotenoid production [6,9]. Both of these goals can be achieved through genomic and functional analyses of psychrotolerant microorganisms from remote regions, like Antarctica, that can be an endless source of biotechnologically valuable organisms.

Bacteria of the genus *Planococcus* are aerobic, Gram-positive, motile cocci belonging to the *Micrococcae* family. These bacteria are common in various environments, often including extreme ones, such as the psychrosphere. Among *Planococcus* species, certain strains were recognized as producers of rare C_30_ carotenoids. However, there remains relatively little information regarding the biosynthesis pathway of C_30_ carotenoids. On the other hand, these carotenoids are promising factors influencing stem cell proliferation, and they possess considerable antioxidative activity, which increases the attractiveness of these metabolites in biotechnology [10,11,12]. *Planococcus* spp. were also used as carotenoid producers from waste materials, like cellulose pulp, which may lower pigment production costs [13].

Another bacterial group that is interesting from the perspective of carotenoid production is *Rhodococcus* spp. Rhodococci are aerobic, Gram-positive, and nonmotile actinomycetes that belong to the *Nocardiaceae* family [14]. Bacteria from the *Rhodococcus* genus are very common in many environments and, similarly to the above mentioned *Planococcus* spp., they are common in extremely cold regions [15]. The metabolic abilities of the plethora of *Rhodococcus* species allow them to use many waste materials (e.g., lube oil [16], corn stover [17], or fruit pulp and peels [18]) as a carbon source during carotenoids production [19,20].

In this study, we performed a thorough genomic analysis of two Antarctic bacteria, *Planococcus* sp. ANT_H30 and *Rhodococcus* sp. ANT_H53B, which were recognized as potential carotenoid producers. We aimed to identify the metabolic pathways responsible for carotenoid production that can be coupled with the results of biochemical analyses of the produced pigments. We tested the ability of both strains to produce carotenoids using waste materials.

## 2. Results and Discussion

### 2.1. General Physiological Characterization of Planococcus sp. ANT_H30 and Rhodococcus sp. ANT_H53B

**Figure 1:**
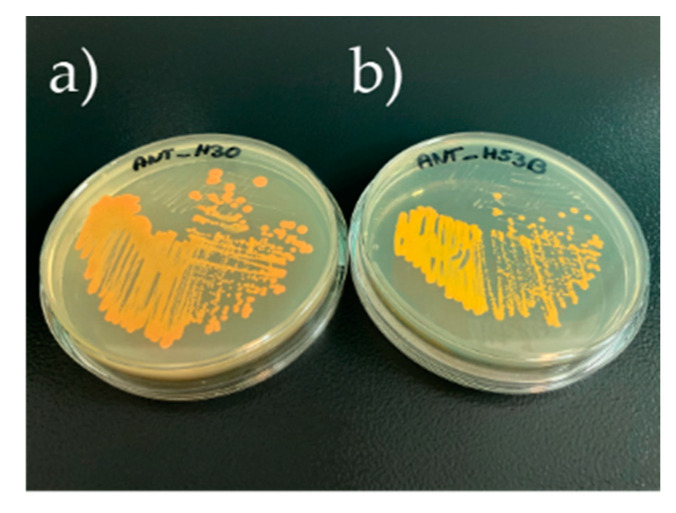
Orange pigmented Antarctic strains analyzed in this study: (**a**) *Planococcus* sp. ANT_H30 and (**b**) *Rhodococcus* sp. ANT_H53B. Bacteria were cultivated on agar-solidified lysogeny broth (LB) medium.

Both strains, i.e., *Planococcus* sp. ANT_H30 and *Rhodococcus* sp. ANT_H53B, originated from a collection of bacterial cultures that were previously isolated from soil samples taken in 2012 from King George Island (Antarctica, GPS coordinates: 62°09.601′ S, 58°28.464′ W) [21]. The strains are orange pigmented (Figure 1), which suggested that they are carotenoid producers. The ANT_H30 strain is able to grow in a wide range of temperatures, ranging from 4 to 37 °C, tolerates pH between 4 and 11, and is halotolerant, as it tolerates NaCl of up to 6%. *Rhodococcus* sp. ANT_H53B is able to grow in temperatures from 4 to 30 °C, it tolerates pH ranging between 5 and 12, and is also halotolerant (tolerates up to 6% salinity). Besides carotenoids, both strains were screened for the production of other secondary metabolites. This demonstrated that ANT_H30, as well as ANT_H53B, possess the ability to produce siderophores or other compounds scavenging iron (Supplementary Figure S1). Additionally, in the case of ANT_H53B, the ability to produce surface-active compounds was detected. Bacteria cultivated in the lysogeny broth (LB) medium with the addition of vegetable oil (1% *w/v*) lowered the interfacial tension (IFT) up to 25% (from 55 (±2) to 40 (±2) mN/m).

### 2.2. Genomic Characterization of Bacterial Strains and Identification of Carotenoid Biosynthesis Gene Clusters

#### 2.2.1. Genomes Sequencing and Overall Genomic Characterization

Sequencing of the *Planococcus* sp. ANT_H30 and *Rhodococcus* sp. ANT_H53B genomes using the Illumina MiSeq platform generated 3,109,614 paired-reads and 932,291,246 nucleotides and 4,432,282 paired-reads and 1,333,091,924 nucleotides, respectively. As a result of the assembly of the *Planococcus* sp. ANT_H30 genome, 22 contigs of a total length of 3,636,638 bp were obtained. In the case of the assembly of the *Rhodococcus* sp. ANT_H53B genome, 37 contigs of a total length of 5,176,448 bp were generated. The genome sequences were initially automatically annotated using RAST on the PATRIC 3.6.2 web service, and the general features for both strains are presented in Table 1.

#### 2.2.2. Identification of Carotenoids Biosynthesis Gene Clusters

**Figure 2:**
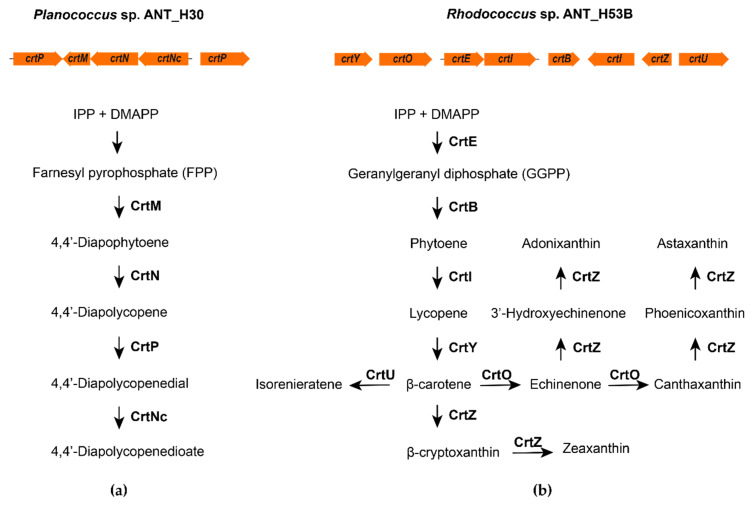
Genes and predicted carotenoid biosynthesis pathways of (**a**) *Planococcus* sp. ANT_H30 and (**b**) *Rhodococcus* sp. ANT_H53B. The following abbreviations mean: IPP, isopentenyl pyrophosphate; DMAPP, dimethylallyl pyrophosphate; CrtP, diapolycopene oxygenase; CrtM, dehydrosqualene synthase; CrtN, dehydrosqualene desaturase; CrtNc, 4,4′-diapolycopene oxidase; CrtE, geranylgeranyl diphosphate synthase; CrtY, lycopene beta-cyclase; CrtO, beta-carotene ketolase; CrtI, phytoene dehydrogenase; CrtB, phytoene synthase; CrtZ, carotene hydroxylase; CrtU, phi-carotenoid synthase.

Genomic analyses of *Planococcus* sp. ANT_H30 and *Rhodococcus* sp. ANT_H53B allowed the identification of genes related to the biosynthesis of carotenoids, i.e., *crt* genes (Table 2). In the ANT_H30 genome, the following genes were identified as a clustered unit—*crtP*, *crtM*, *crtN*, *crtNc,* and a single unclustered *crtP* gene (a second copy of *crtP*; Figure 2). These genes are crucial in the production of C_30_ apocarotenoids, like 4,4′-diapolycopene and its derivatives (Figure 2), and are common in *Planococcus* species [22]. Apocarotenoid-producers are relatively rare among bacteria [23].

Within the genome of the ANT_H53B, we found eight *crt* genes, i.e., *crtE*, *crtY*, *crtO*, *crtI* (two copies), *crtB*, *crtZ*, and *crtU*. (Figure 2 and Table 2). The ANT_H53B strain possessed the *crtE* gene, which encodes geranylgeranyl diphosphate synthase, which is essential in the synthesis of C_40_ carotenoids. In the case of ANT_H53B, as well as other rhodococci [20], the end products of the carotenoid biosynthetic pathway may potentially include various carotenoids or xanthophylls, including: echinenone, astaxanthin, hydroxyechineone, cryptoxanthin, chlorobactene, and isorenieratene (Figure 2).

#### 2.2.3. Genome-Based Insight into the Metabolic Potential

Our analysis of the metabolic potential of the *Planococcus* sp. ANT_H30 strain revealed various metabolic modules of the carbohydrate metabolism, including the Embden–Meyerhof pathway of glycolysis, gluconeogenesis, the citrate cycle (Krebs cycle), the nonoxidative phase pentose phosphate pathway, and the Leloir pathway of galactose degradation. The ANT_H30 strain was shown to possess genes encoding enzymes involved in the degradation of fatty acids, ketone bodies, and amino acids, like valine, leucine, isoleucine, and lysine. Within the genome of *Planococcus* sp. ANT_H30, as well as other *Planococcus* strains (i.e., S5 [24], ZD22 [25], and PAMC21323 [26]), there are single genes (but not full pathways) encoding enzymes involved in the meta-cleavage of catechol compounds (catechol 2,3-dioxygenase (EC: 1.13.11.2) (GenBank accession number: FQ085_09110)) and in the degradation of several xenobiotics, such as benzoates and xylene (3-oxoadipate enol-lactonase (EC: 3.1.1.24) (GenBank accession number: FQ085_11270), 4-oxalocrotonate tautomerase (EC: 5.3.2.6) (GenBank accession number: FQ085_03925)), aminobenzoates and styrene (amidase (EC: 3.5.1.4) (GenBank accession number: FQ085_10470), 4-nitrophenyl phosphatase (EC: 3.1.3.41) (GenBank accession number: FQ085_11855)), ethylbenzene (acetyl coenzyme A (acetyl-CoA) acyltransferase (EC: 2.3.1.16) (GenBank accession number: FQ085_08820 and FQ085_11980)), and naphthalene (alcohol dehydrogenase (EC: 1.1.1.1) (GenBank accession number: FQ085_04180 and FQ085_12880)).

Some enzymes that take part in the degradation of halogenated compounds, such as dioxins, chloroalkanes, chloroalkenes, chlorocyclohexane, and chlorobenzene (e.g., aldehyde dehydrogenase (NAD^+^) (EC: 1.2.1.3) (GenBank accession number: FQ085_01960 and FQ085_11355), formaldehyde dehydrogenase (EC: 1.2.1.46) (GenBank accession number: FQ085_14150), and 2-haloacid dehalogenase (EC: 3.8.1.2) (GenBank accession number: FQ085_08115)). In the genome of ANT_H30, cyanate lyase (EC: 4.2.1.104) (GenBank accession number: FQ085_05675), which participates in the breakdown of harmful cyanides into carbamate [27], and nitrilase (EC: 3.5.5.1) (GenBank accession number: FQ085_05485), which catalyses the hydrolysis of nitrile compounds [28], are also encoded. These enzymes take part in the degradation of toxic compounds into carboxylic acid and ammonia, which may constitute a potential source of carbon and nitrogen.

The potential for utilization of various complex compounds and their usage as carbon and/or nitrogen sources is a huge advantage of using by-products or waste products for strain cultivation and desired metabolite production. In the genome of *Planococcus* ANT_H30, a number of genes related to siderophores synthesis were also found. Despite several ATP-binding cassette (ABC)-type Fe^3+^-siderophore transport systems (GenBank accession number: FQ085_00085, FQ085_13725, and FQ085_13730) there were genes encoding proteins responsible and crucial for staphylobactin-like siderophore production—SirA (GenBank accession number: FQ085_00100), SirB (FQ085_00095), and SirC (GenBank accession number: FQ085_00090).

We also analysed the metabolic potential of *Rhodococcus* sp. ANT_H53B. The genetic modules responsible for the basic carbohydrate metabolism were similar to ANT_H30, i.e., the Embden–Meyerhof pathway of glycolysis, gluconeogenesis, the citrate cycle (Krebs cycle), the non-oxidative phase pentose phosphate pathway, and the Leroir pathway of galactose degradation. Genes related with the oxidative phase pentose phosphate pathway, glycogen degradation, and propanoyl-CoA metabolism, as well as the degradation of fatty acids, ketone bodies, and acylglycerol (triacylglycerol lipase (EC: 3.1.1.3) (GenBank accession number: FQ188_10910)), were also present in the genome of ANT_H53B. Degradation pathways of amino acids, like valine, leucine, isoleucine, and lysine, were present.

Deeper genomic analyses revealed predicted abilities of ANT_H53B to obtain energy from dissimilatory nitrate reduction (i.e., nitrite reductase (NADH) (EC: 1.7.1.15) (GenBank accession number: FQ188_04565 and FQ188_06435)) and assimilatory sulfate reduction (i.e., phosphoadenosine phosphosulfate reductase (EC: 1.8.4.8) (GenBank accession number: FQ188_09865), sulfite reductase (ferredoxin) (EC: 1.8.7.1) (GenBank accession number: FQ188_09860), and sulfite reductase (NADPH) flavoprotein (EC: 1.8.1.2) (GenBank accession number: FQ188_10550 and FQ188_20710), which is common within *Rhodococcus* species (e.g., RB1 [29] and Eu-32 [30]). ANT_H53B possessed genes enabling the synthesis of cofactor F420 (i.e., 7,8-didemethyl-8-hydroxy-5-deazariboflavin (FO) synthase (EC: 4.3.1.32) (GenBank accession number: FQ188_19600), 2-phospho-l-lactate guanylyltransferase (EC: 2.7.7.68) (GenBank accession number: FQ188_05230), FO 2-phospho-l-lactate transferase (EC: 2.7.8.28) (GenBank accession number: FQ188_04305), and F420-0:l-glutamate ligase (EC: 6.3.2.31) (GenBank accession number: FQ188_04300)), involved in catalyzing a wide range of complex enzymatic redox reactions, which are widely distributed amongst archaeal methanogens and actinomycetes, including rhodococci [31,32].

*Rhodococcus* sp. ANT_H53B, as well as other described strains of this genus, e.g., P14 [33], 17895 [34] and RKJ300 [35], possess a wide spectrum of genes involved in the degradation of numerous hydrocarbons and xenobiotics. Genome analysis indicated the ability to degrade:

(i) benzoates and ethylbenzene—using e.g., 4-methoxybenzoate monooxygenase (EC: 1.14.99.15) (GenBank accession number: FQ188_15500), *P*-hydroxybenzoate 3-monooxygenase (EC: 1.14.13.2) (GenBank accession number: FQ188_16125), benzoate 1,2-dioxygenase (EC: 1.14.12.10) (GenBank accession number: FQ188_16155), protocatechuate 3,4-dioxygenase (EC: 1.13.11.3) (GenBank accession number: FQ188_10175), and hydroxyquinol 1,2-dioxygenase (EC: 1.13.11.37) (GenBank accession number: FQ188_18125);
(ii) aminobenzoates—with the use of amidase (EC: 3.5.1.4) (GenBank accession numbers: FQ188_09805, FQ188_11165, and FQ188_16180), and monooxygenase (EC: 1.14.13.-) (GenBank accession numbers: FQ188_11040 and FQ188_17850);
(iii) fluorobenzoates—using carboxymethylenebutenolidase (EC: 3.1.1.45) (GenBank accession numbers: FQ188_13875, FQ188_14120, FQ188_15865, and FQ188_18215);
(iv) toluene and xylene—using benzaldehyde dehydrogenase (EC: 1.2.1.28) (GenBank accession number: FQ188_11010), maleylacetate reductase (EC: 1.3.1.32) (GenBank accession number: FQ188_18130), and catechol 1,2-dioxygenase (EC: 1.13.11.1) (GenBank accession number: FQ188_16170);
(v) nitro compounds, such as nitrotoluene, atrazine, caprolactam—using dihydropteridine reductase (EC: 1.5.1.34) (GenBank accession number: FQ188_00250), and *N*-ethylmaleimide reductase (GenBank accession number: FQ188_00250);
(vi) halogenated compounds, like dioxins, chloroalkanes, chloroalkenes, chlorocyclohexane, and chlorobenzene—using 2-haloacid dehalogenase (EC: 3.8.1.2) (GenBank accession numbers: FQ188_04145, FQ188_19570, and FQ188_19725), 2,4-dichlorophenol 6-monooxygenase (EC: 1.14.13.20) (GenBank accession numbers: FQ188_15475 and FQ188_18135).

In the genome of ANT_H53B, there were also genes indicating the ability to synthesize and transport siderophores, including several ABC-type Fe^3+^-siderophore transport systems (GenBank accession numbers: FQ188_00500, FQ188_00505, FQ188_00510, FQ188_06045, FQ188_06050, and FQ188_06055), siderophore monooxygenase (GenBank accession number: FQ188_00495), and modules of non-ribosomal peptide synthetase (GenBank accession numbers: FQ188_00615, FQ188_03130, FQ188_03135, FQ188_03140, and FQ188_06210). ANT_H53B possesses genes responsible for the synthesis of trehalose-derived surfactants, typical for *Rhodococcus* species [36], i.e., trehalose-6-phosphate phosphatase (EC: 3.1.3.12) (GenBank accession numbers: FQ188_11590, FQ188_17500) and trehalose O-mycolyltransferase (EC 2.3.1.122) (GenBank accession numbers: FQ188_20530, FQ188_20535, and FQ188_20540), which increases the availability and, thus, utilization of hydrophobic substrates.

#### 2.2.4. Biosafety Considerations of *Planococcus* sp. ANT_H30 and *Rhodococcus* sp. ANT_H53B

The *Planococcus* and *Rhodococcus* species are very common in various environments. So far, no pathogens from the genus *Planococcus* have been reported, while, within the genus *Rhodococcus,* two pathogenic species, *R. fascians* and *R. equi*—infecting plants [37] and mammals [38], respectively—were found. As both strains analyzed in this study can be potentially applied in biotechnological processes, it is necessary to analyse their biosafety. The genomic analysis of *Planococcus* sp. ANT_H30 genome using the Resistance Gene Identifier (RGI) analyzer indicated the absence of antibiotic resistance genes, while in the case of *Rhodococcus* sp. ANT_H53B, this revealed the presence of a putative rifampicin resistance gene, *RbpA* (encoding RNA polymerase (RNAP)-binding protein, whose presence increased the tolerance levels of mycobacteria to rifampicin by an unknown mechanism) with 100% similarity to protein WP_027497120 [39]. To determine whether the predicted antibiotic resistance gene was truly associated with the resistance phenotype, we tested the MIC (minimum inhibitory concentration) of rifampicin resistance. The result revealed the sensitivity to the tested antibiotic (MIC was lower than 0.016 mg/L of rifampicin concentration).

In addition, single genes potentially related to virulence were detected in the genomes of both strains. In the genome of ANT_H30, there was a *clpP* gene (GenBank accession number: FQ085_12085) that encodes the ATP-dependent Clp protease proteolytic subunit of ClpP (EC: 3.4.21.92). Clp proteolytic complexes are responsible for the adaptation of bacteria to stress by degrading accumulated and misfolded proteins but were also reported as putative virulence factors [40]. In the case of ANT_H53B, the *icl* gene (GenBank accession number: FQ188_12695) was identified as a virulence factor. The *icl* gene encodes isocitrate lyase (EC: 4.1.3.1), which is an important virulence factor of, e.g., pathogenic *R. equi*. Isocitrate lyase contributes to the acquisition of membrane lipid-derived fatty acids [41].

### 2.3. Chemical Identification of Synthesized Carotenoids

The ultraperformance liquid chromatography (UPLC) analyses of the carotenoid extract from the ANT_H30 strain revealed the presence of two potential carotenoids. They demonstrated relatively large masses, i.e., 857.5498 and 871.5688 Da, which cannot be assigned to any known carotenoids. However, they have the spectra characteristics of carotenoids (289 nm; 468 nm; 495 nm and 289 nm; 468 nm; 494 nm; Supplementary Table S2). The inability to precisely identify carotenoids from ANT_H30 extract may be due to the ability of carotenoids to aggregate or form connections with lipids [42]. *Planococcus* sp. ANT_H30 is an Antarctic strain, and the specific placement of carotenoids for cryoprotective purposes may contribute to significant modifications in the structure of carotenoids [4]. Therefore, it is possible that novel carotenoids were found; however, this requires further investigation.

For ANT_H53B, a mixture of six different carotenoids was identified (Supplementary Table S2). Among them, we accurately determined the presence of two compounds, i.e., dihydroxyneurosporene and hydroxyechinenone, and this was also partially confirmed by genomic analyses. As a result of the gas chromatography–mass spectrometry (GC-MS) analysis, mass ions of 587.91, 575.46, and 577.48 Da with retention times of 27.994, 33.311, and 33.121 min, respectively (Supplementary Figure S2), were present in the carotenoid extract from ANT_H53B. This result confirmed the presence of dihydroxyneurosporene (575.4602 Da) and two other (not identified by name) carotenoids (i.e., 587.9095 and 577.4785 Da) found using UPLC. For ANT_H53B, the presence of carotenoid with a mass of 536.87 Da, correlated with lycopene or beta-carotene mass was also confirmed.

### 2.4. Free Radical Scavenging Activity

One of the main advantages of carotenoids is their distinctive, polyunsaturated chemical formula. This feature determines their ability to scavenge free radicals, and thus carotenoids constitute the desired metabolite in the food, pharmaceutical, and medicine industries. The antioxidant potencies of the ANT_H30 and AND_H53B carotenoid extracts were evaluated by the DPPH (2,2-diphenyl-1-picrylhydrazyl) method [43], which enabled us to measure the free radical scavenging ability.

**Figure 3:**
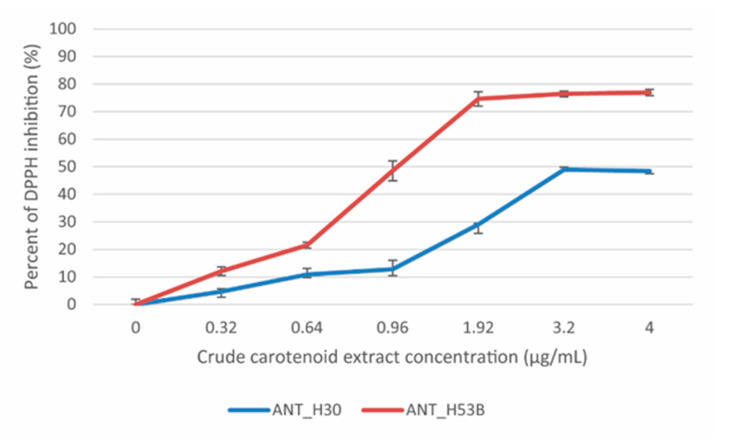
DPPH inhibition test performed using the ANT_H30 and ANT_H53B crude carotenoid extracts. Error bars represent standard deviations of the triplicates.

The scavenging effect was tested on a 0.1 mM DPPH solution (used as a free radical) and revealed the IC_50_ values (antioxidant concentration required for quenching 50% of the initial DPPH) were 3.2 µg/mL and 0.96 µg/mL for the extracts obtained from ANT_H30 and ANT_H53B, respectively. It was shown that ANT_H30 quenched 50% and ANT_H53B 77% of the DPPH maximally (Figure 3). These results indicate a high antioxidant capacity of both crude carotenoid extracts; however, the higher scavenging activity was proven for carotenoids produced by ANT_H53B.

### 2.5. Optimization of Production of Carotenoids

**Figure 4:**
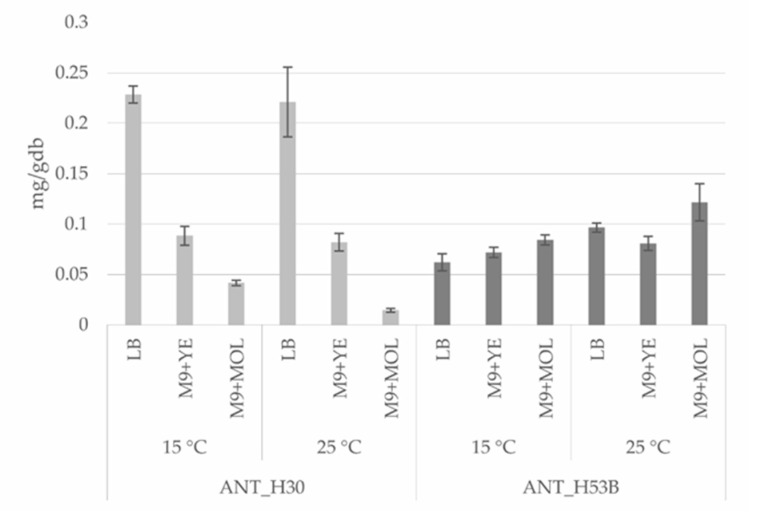
The quantity of extracted crude carotenoids from *Planococcus* sp. ANT_H30 and *Rhodococcus* sp. ANT_H53B cultivated in various media and at various temperatures.

The optimization of the production of carotenoids in the ANT_H30 and ANT_H53B strains was performed using various growth media (including minimal medium supplemented with cheap industrial byproducts, i.e., molasses and yeast extract) and at various temperatures (Figure 4).

After four days of cultivation of ANT_H30 at 15 °C in LB medium, the bacteria produced 0.228 mg (±0.008) of crude carotenoid extract per gram of dry biomass (gdb). On the other hand, this strain also synthesized a significant amount of carotenoids on M9 (minimal) medium supplemented with yeast extract (M9 + YE). In this case, the result was 0.088 mg (±0.009)/gdb after four days of cultivation. Although this result is almost 2.5-times lower when compared with the cultivation on LB medium, it should be emphasized that yeast extract is an easily accessible industrial byproduct. However, ANT_H30 cultivated in M9 medium supplemented with molasses (M9 + MOL) in 15 °C produced only 0.042 mg (±0.003) of carotenoids/gdb after four days of cultivation, which is almost two-times lower than in the case of M9 + YE and as much as 5.5-times lower than for LB cultures.

The ANT_H30 strain is a psychrotolerant, however, also demonstrated good growth at higher temperatures. In order to determine the carotenoid production efficiency at higher temperatures, ANT_H30 was cultivated at 25 °C. This revealed a similar capacity to produce biomass and carotenoids as at 15 °C. After four days of cultivation of ANT_H30 in LB and M9 + YE at 15 °C, the amounts of carotenoids reached, respectively, 0.221 mg (±0.035)/gdb and 0.082 mg (±0.009)/gdb. However, in the case of M9 + MOL, the amount was significantly lower (0.015 (±0.002)/gdb). This result suggests that ANT_H30 can be cultivated and potentially used for carotenoids production, at low temperatures, which may reduce the overall costs of pigment biosynthesis.

The results for the ANT_H53B strain differed significantly. After four days of cultivation in LB medium at 15 °C, the amount of crude carotenoid extract reached 0.062 mg (±0.008)/gdb. A similar result was obtained for ANT_H53B cultivated in M9 + YE, i.e., 0.072 (±0.005)/gdb. The best conditions for carotenoid production were obtained when cultivating in M9 + MOL medium at 15 °C. After four days of cultivation, the amount of desired metabolites was 0.084 mg (±0.005)/gdb. The production of carotenoids by ANT_H53B at a higher temperature (25 °C) resulted in similar amounts, i.e., 0.1 mg (±0.005)/gdb and 0.08 mg (±0.007)/gdb for LB and M9 + YE cultures, respectively. A significant increase of carotenoid production was achieved for cultures in M9 + MOL at 25 °C and the amount of orange pigments reached 0.122 mg (±0.018)/gdb.

Asker et al. [44] determined the production of carotenoids (per gram of dry biomass) from a number of mesophilic bacterial strains belonging to the classes *Flavobacteria*, *Sphingobacteria*, *Alphaproteobacteria*, *Gammaproteobacteria*, *Actinobacteria*, *Bacilli,* and *Deinococci*. Among 104 strains of carotenoid-producing bacteria, only a few isolates were recognized as efficient carotenoid producers, i.e., *Pedobacter* sp. TDMA-5 (0.8 mg/gdb), *Brevudimonas* sp. TDMA-7 (1.4 mg/gdb), *Paracoccus* sp. TDMA-8 (1.1 mg/gdb), and two strains of *Sphingomonas* genus—TDMA-16 (1.7 mg/gdb) and TDMA-17 (2.8 mg/gdb). On the other hand, Vila et al. [45] examined strains isolated from King George Island for carotenoid production. In this study, the production of carotenoids oscillated around 0.5 mg/gdb for *Planococcus* sp. P48, while for *Arthrobacter* sp. P40 and *Cryobacterium* sp. P19 it was around 0.3 mg/gdb and 0.4 mg/gdb, respectively.

Currently, in industry, two groups of strains are used for the production of carotenoids, i.e., natural (unmodified, environmental isolates) and genetically modified strains. These genetically modified strains are able to produce a much higher amount of carotenoids (e.g., around 12 mg/gdb carotenoids from the modified *Escherichia coli* [46]). However, these are genetically modified strains and their usage may be restricted. As for the natural, environmental isolates used in an industry this production yield is usually much lower. For example, *Brevundimonas* sp. N-5, recognized as a very efficient producer of carotenoids, synthesized around 0.6 mg/gdb [47]. Therefore, the strains ANT_H30 and ANT_H53B, described in this study, may be recognized as moderately efficient producers of carotenoids. However, a considerable advantage of both strains is their ability to produce biomass (and carotenoids) at low temperature, which may significantly reduce the costs of pigments production. Additionally, we proved that they can use cheap waste products (e.g., molasses) for the production of biomass, which also may further reduce the production costs.

## 3. Materials and Methods

### 3.1. Bacterial Strains and Culture Conditions

The bacterial strains were cultivated in lysogeny broth (LB) and minimal medium M9 [48], supplemented (0.5% (*w/v*)) with various carbon sources (i.e., yeast extract and beet molasses) at 15 °C and 25 °C with rotary shaking set to 150 rpm. The growth kinetics were assessed by measuring the changes in the optical density of cultures in comparison with the noninoculated controls, using an automated microplate reader (Sunrise TECAN, Tecan Trading AG, Männedorf, Switzerland). Before inoculation of the supplemented M9 medium, the bacteria were cultivated overnight in LB medium at the optimal growth temperature (15 °C). Overnight cultures were then centrifuged (6000 rpm for 5 min) and washed three times with 0.85% saline solution. Next, the bacteria were diluted in triplicate, into the fresh M9 medium supplemented with an appropriate carbon source. In each case, the initial optical density at 600 nm (OD_600_) was 0.05. The OD_600_ of the respective cultures was measured every 24 h for five days.

### 3.2. Detection of Siderophores and Surfactants

To determine the ability to produce siderophores and/or other iron scavenging compounds, bacteria were cultivated on the GASN medium [49] for 4 days at 20 °C with rotary shaking set to 150 rpm. Bacteria were then centrifuged (6000 rpm/5 min) and the obtained supernatants were added in a 1:1 ratio to the CAS reagent [50]. GASN medium was used as a negative control, while deferoxamine mesylate salt (Sigma-Aldrich Co., St. Louis, MO, USA), at a concentration of 0.025 mM, was used as a positive control. All experiments were performed in triplicates. After an hour of incubation, the absorbance at 630 nm was measured using an automated microplate reader (Sunrise TECAN; Supplementary Figure S1).

### 3.3. Draft Genome Sequencing

Genomic DNAs of the *Planococcus* sp. ANT_H30 and *Rhodococcus* sp. ANT_H53B were isolated using the CTAB (cetyl trimethylammonium bromide)/lysozyme) method [51]. An Illumina TruSeq library was constructed following the manufacturer’s instructions. The genomic libraries were sequenced on an Illumina MiSeq instrument (using the v3 chemistry kit; Illumina, San Diego, CA, USA) in the DNA Sequencing and Oligonucleotide Synthesis Laboratory (oligo.pl) at the Institute of Biochemistry and Biophysics, Polish Academy of Sciences, Warsaw. The reads trimmed with CutAdapt v. 1.9.1 [52] were further assembled using Newbler De Novo Assembler v. 3.0 (Roche, Basel, Switzerland).

### 3.4. Bioinformatics

The *Planococcus* sp. ANT_H30 and *Rhodococcus* sp. ANT_H53B genomes were manually annotated using the MAISEN platform and automatically annotated using RAST [53] on the PATRIC 3.6.2 [54] web service. Similarity searches were performed using BLAST programs [55]. The metabolic features were identified with the SEED viewer web server [56], KEGG (Kyoto Encyclopedia of Genes and Genomes) Automatic Annotation System (KAAS) database [57], and the bacterial version of the antiSmash web server [58]. All options were selected with the default parameters. Additionally, for deeper metabolic investigation, the amino acid sequences were subjected to BLAST-KOALA analysis [59]. The KO (KEGG Orthology) assignments were performed using a modified version of the internally used KOALA (KEGG Orthology And Links Annotation) algorithm (BLAST-KOALA) after the BLAST search against a nonredundant dataset of pangenome sequences [59]. To investigate the virulence factors of the tested strains, the VFDB database (Virulence Factors Database) was used [60].

To identify putative antibiotic resistance genes, we used the Resistance Gene Identifier (RGI) in the Comprehensive Antibiotic Resistance Database (CARD) [61] software. Hits showing at least 50% identity with the reference protein were considered significant. Each hit was verified manually using BLASTp analysis.

### 3.5. Antibiotic Susceptibility Testing

To determine the rifampicin susceptibility pattern of tested bacteria, the MIC of this antibiotic was assessed using Etest™ (Liofilchem, Roseto degli Abruzzi, Italy). The analysis was conducted according to the European Committee on Antimicrobial Susceptibility Testing (EUCAST) recommendations [62].

### 3.6. Extraction of Carotenoids from Bacterial Culture

The extraction of nonpolar lipids from *Planococcus* sp. ANT_H30 and *Rhodococcus* sp. ANT_H53B was conducted in a climate room at 4 °C. We transferred 0.5 mL of a centrifuged suspension of bacteria to a 45 mL Corex tube and, after the addition of 4 mL of methanol, sonicated the sample with an ultrasonic cleaner (Sharpertek^®^, Pontiac, MI, USA) for 15 min. Then, another 5 mL of methanol was added and the sample was shaken in a reciprocating shaker (PROMAX 2020, Heidolph, Germany) for 15 min. After shaking, the sample was stored without agitation in the refrigerator (−12 °C) for 15–20 min to allow the dregs to descend. The clear methanol phase was transferred to another Corex tube, and the whole process was repeated until the bacterial suspension was discolored (typically 2–3 times). The combined methanol extracts were filtered through a Millipore syringe filter unit Millex-CV13 Filter Unit (0.22 μm), evaporated to dryness under argon at 35 °C and dissolved in 1 mL methanol-propanol-hexane 6:1:3 (*v/v/v*) mixture. Before the UPLC analysis, the samples were stored under argon at −70 °C.

### 3.7. Qualitative Analysis of Carotenoids

#### 3.7.1. Ultraperformance Liquid Chromatography (UPLC)

The carotenoid compositions were analyzed by a modified method described previously [63]. The extracted pigments were separated using the Acquity Ultra Performance LC system (Waters, Milford, MA, USA) connected with the Synapt G2 HDMS mass spectrometer (Waters). The samples were injected (7.5 μL) into an Acquity UPLC HSS T3 (1.8 mm, 1.0 × 150 mm) analytical column. Initially, the column was eluted at 25 °C at a constant flow rate of 35 μL/min with 100% of solvent A (water-methanol 15:85, *v/v*) and, after injection at the same condition, for the next 15 min. Next, the stepped linear gradient of buffer B (methanol/2-propanol/hexane 2:1:1, *v/v*) was distributed as follows: 0–15% B at 15–160 min (flow rate = 35 μL/min); 15%–80% B at 160–240 min (flow rate = 35–20 μL/min); 80%–90% B at 240–245 min (flow rate = 20 μL min); 90%–100% B at 245–255 min (flow rate = 20–80 μL/min); and held for 10 min at 100% buffer B. Within the next 5 min, the concentration of solvent B was decreased to 0% and the column was equilibrated for 8 min at a flow rate of 80 μL/min and 7 min at a flow rate from 80 to 5 μL/min before the next injection.

The separation of components was monitored using a photodiode array detector at the 200–750 nm range and a mass spectrometer at the 100–1000 *m*/*z* range. A positive electrospray ionization mode (ES+) with a TOF detector was used. The identification of components was performed by the analysis of the absorbance spectra in connection with mass spectra (Supplementary Figure S2) with the use of MassLynx 4.1 software (Waters). The chromatograms were presented at the wavelength characteristic for carotenoids (470 nm) and at the appropriate mass of identified components (Supplementary Figure S2).

#### 3.7.2. Gas Chromatography-Mass Spectrometry (GC-MS)

The separation of single mass was performed using an Agilent 7890A Series Gas Chromatograph interfaced to an Agilent 5973c Network Mass Selective Detector and an Agilent 7683 Series Injector (Agilent Technologies, Palo Alto, CA, USA). A 5 µL sample was injected with split 1:5 (sample/carrier gas) to an HP-5MS column (30 m × 0.25 mm I.D., 0.25 µm film thickness) using He as the carrier gas at 1 mL min^−1^. The ion source was maintained at 250 °C; the GC oven was programmed with a temperature gradient starting at 50 °C (for 8 min) and this was gradually increased to 325 °C (for 10 min) at 7 °C min^−1^. Mass spectrometry analysis was conducted in the electron-impact mode at an ionizing potential of 70 eV. The mass spectra were recorded from the selected ion monitoring (SIM) mode. In the SIM mode, the GC-MS collected signals from the individual ions.

### 3.8. Quantitative Analysis of Carotenoids

Bacterial strains were cultivated in lysogeny broth (LB) and minimal medium M9 [48], supplemented (0.5% (*w/v*)) with beet molasses or yeast extract for four days in two different temperatures, i.e., 15 °C and 25 °C. Both bacterial strains were cultivated in an initial volume of 100 mL in triplicate. Every day, triplicates of each strain were divided into two equal parts. Part of the culture (i.e., 50 mL) was extracted and then measured spectrophotometrically using Evolution 260 Bio (Thermo Fisher Scientific, Waltham, MA, USA) to establish the maximum absorbance value. To determine the concentration of the crude carotenoid extract, the method of Liaaen-Jensen and Jensen was used [62]. The bacterial pellet was resuspended in 10 mL of acetone-methanol (7:2 *v/v*) solution, sonicated at ultrasonic cleaner for 5 min and filtered through Whatman filter paper (Whatman, Maidstone, United Kingdom). The absorbance of this extracted solution was measured spectrophotometrically at 453 nm and calculated according to Jensen’s equation [62]. The remaining half of bacterial cultures (i.e., 50 mL) was dried at 100 °C for 24 h and weighed to obtain the dry mass weight information.

### 3.9. Free Radical Scavenging Activity

The DPPH (2,2-diphenyl-1-picrylhydrazyl) method was used to measure the free radical scavenging activity. The dilutions were prepared as followed: 2 mL of 0.1 mM DPPH in methanol was added to 2 mL of methanol containing different amounts of crude carotenoid extracts of ANT_H30 or ANT_H53B, i.e., to reach final concentrations of: 0.32, 0.64, 0.96, 1.92, 3.2, and 4 ug/mL of carotenoids. The absorbance at 517 nm was measured spectrophotometrically (Evolution 260 Bio (Thermo Fisher Scientific)) after 30 min. The scavenging of the DPPH radical (%) was calculated according to the formula ((A_0_ − A_1_)/A_0_ × 100), where A_0_ is the absorbance of the control reaction and A_1_ is the absorbance of reactions containing crude carotenoid extract from ANT_H30 or ANT_H53B [64].

### 3.10. Nucleotide Sequence Accession Numbers

The nucleotide sequences of the draft genomes of *Planococcus* sp. ANT_H30 and *Rhodococcus* sp. ANT_H53B were deposited in the GenBank (NCBI) database with the accession numbers NZ_VOBJ00000000 and NZ_VOBD00000000, respectively.

## 4. Conclusions

Two Antarctic bacteria, *Planococcus* sp. ANT_H30 and *Rhodococcus* sp. ANT_H53B, were recognized as carotenoid producers. In-depth genomic and functional analyses revealed that these bacteria may use various compounds as a source of carbon and energy, including xenobiotics and waste materials from industrial production, e.g., molasses or yeast extract. Further genomic analyses identified the *crt* genes responsible for carotenoid biosynthesis. In the genome of ANT_H30, a gene cluster associated with the production of apocarotenoids was identified, while, in ANT_H53B, we found various *crt* genes enabling the biosynthesis of C_40_ carotenoids, such as lycopene, β-carotene, chlorobactene, and astaxanthin. Quantitative analyses of the produced metabolites indicated the possibility of using these bacterial strains for the production of carotenoids at low temperature, in which mesophilic bacteria are no longer active or produce low biomass. Additionally, it was shown that the produced crude carotenoid extracts had a significant ability to scavenge free radicals, which is meaningful for their possible future applications.

## Figures and Tables

**Table 1:** General features of the *Planococcus* sp. ANT_H30 and *Rhodococcus* sp. ANT_H53B draft genomes.

Feature	Calculation	
Strain	ANT_H30	ANT_H53B
Number of Contigs	22	37
Estimated Genome Size (bp)	3,636,638	5,176,448
GC Content (%)	40.8%	64.87%
Number of Genes	3591	4889
Number of Proteins with Functional Assignments	2562	3379
Number of Proteins with Enzyme Commission (EC) Number Assignments	874	1149
Number of Transfer RNA (tRNA) Genes	59	46
Number of Regulatory RNA Genes	23	12

**Table 2:** Carotenoid biosynthesis enzymes encoded by ANT_H30 and ANT_H53B.

Strain	Gene	GenBank Accession Number	Encoded Protein	Reference Protein	Amino Acids Identity
ANT_H30	*crtP*	FQ085_05070; FQ085_10685	Diapolycopene oxygenase (EC: 1.14.99.44)	AUO94_02190; AUO94_13335	98%; 98%
ANT_H30	*crtM*	FQ085_05075	Dehydrosqualene synthase (EC: 2.5.1.96)	AUO94_02185	98%
ANT_H30	*crtN*	FQ085_05080	Dehydrosqualene desaturase (EC: 1.3.8.2)	AUO94_02180	98%
ANT_H30	*crtNc*	FQ085_05085	4,4′-diapolycopene oxidase (EC: 1.14.99.44)	AUO94_02175	98%
ANT_H53B	*crtE*	FQ188_09125	Geranylgeranyl diphosphate synthase (EC: 2.5.1.29)	NY08_684	95%
ANT_H53B	*crtY*	FQ188_06685	Lycopene beta-cyclase (EC: 5.5.1.19)	NY08_1078	93%
ANT_H53B	*crtO*	FQ188_09100	Beta-carotene ketolase (EC: 1.14.99.63)	NY08_689	95%
ANT_H53B	*crtI*	FQ188_09130, FQ188_15555	Phytoene dehydrogenase (EC: 1.14.99.-)	NY08_683; NY08_2230	94%; 90%
ANT_H53B	*crtB*	FQ188_09140	Phytoene synthase (EC: 2.5.1.32)	NY08_680	95%
ANT_H53B	*crtZ*	FQ188_15555	Carotene hydroxylase (EC: 1.14.13.129)	NY08_4121	95%
ANT_H53B	*crtU*	FQ188_19840	Phi-Carotenoid synthase (EC: 1.3.99.39)	NY08_3769	96%

## Supplementary Materials

The following are available online, Figure S1: Iron scavenging ability of GASN medium (negative control), supernatants obtained from cultures of ANT_H30 and ANT_H53B and deferoxamine mesylate salt (positive control). Figure S2: GC-MS chromatogram of ANT_H53B carotenoid extract with SIM mode set on: (a) 575.46 Da, (b) 577.48 Da, (c) 587.91 Da. Table S1: Summary of carotenoids identification performed using UPLC.
