# Supplementary material for: Genome-Based Insights into the Production of Carotenoids by Antarctic Bacteria, Planococcus sp. ANT_H30 and Rhodococcus sp. ANT_H53B
Source: Molecules. 2020 Sep 23;25(19):4357. doi: 10.3390/molecules25194357 (PMC7582328; doi:10.3390/molecules25194357)
Supplement: Supplementary file 1 [file molecules-25-04357-s001.pdf]

molecules MDPI

Table S1. Summary of carotenoids identification performed using the UPLC.

Strain	Retention (min)	Mass	Spectrum (nm)	Carotenoid	Structure	Theoretical mass (TM)	TM+H	TM+Na	TM+NaH
ANT_H53B	190.258; 191.727	587.9095	453		C40H52O2	564.3967	565.4045	587.3865	588.3943
ANT_H53B	200.649	577.5206	271; 308; 469		C40H64O2	576.4907	577.4985	599.4804	600.4882
ANT_H53B	222.504; 222.676	575.4602	308; 440sh; 466; 490sh	Dihydroxyneurosporene	C40H62O2	574.475	575.4828	597.4647	598.4726
ANT_H53B	224.765; 225.805	601.4796	309; 469; 493sh		C40H56O4	600.4178	601.4257	623.4077	624.4155
ANT_H53B	229.0595	589.4817	307; 471; 493sh	Hydroxyechinenone	C40H54O2	566.4124	567.4202	589.4022	590.41
ANT_H53B	229.0595	577.4785	255; 480		C40H64O2	576.4907	577.4985	599.4804	600.4882
ANT_H30	201.595; 202.0595	857.5498	289; 468; 495						
ANT_H30	206.0595; 207.691	871.5688	289; 468; 494						

* - The "sh" abbreviation mean short hill.

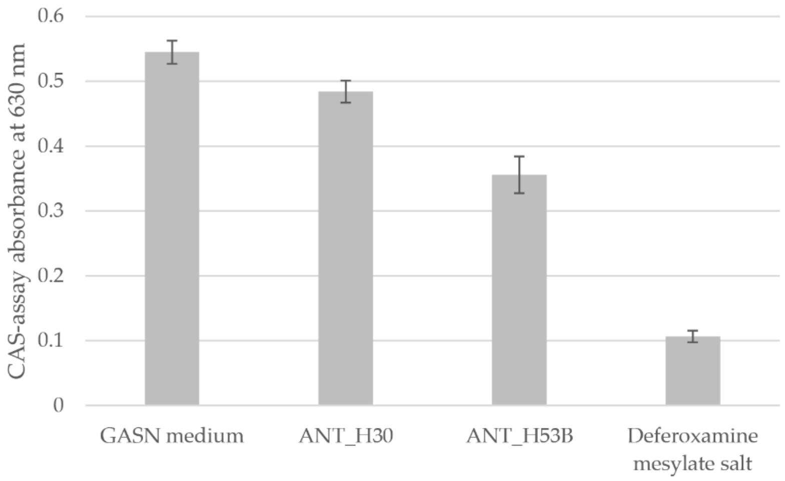

Figure S1. Iron scavenging ability of GASN medium (negative control), supernatant obtained from cultures of ANT_H30 and ANT_H53B and deferoxamine mesylate salt (positive control). Error bars represent standard deviations of the triplicates.

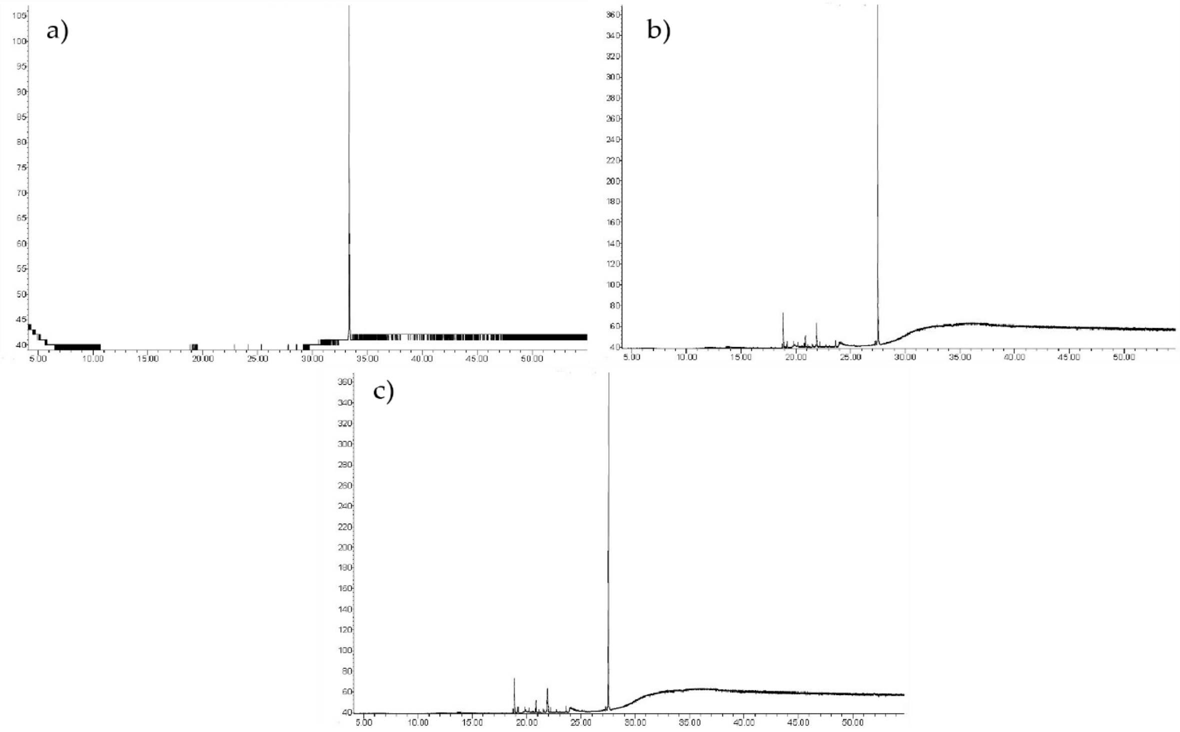

Figure S2. GC-MS chromatogram of ANT_H53B carotenoid extract with SIM-mode set on: a) 575.46 Da, b) 577.48 Da, c) 587.91 Da.
